# Mean deviation based identification of activated voxels from time-series fMRI data of schizophrenia patients

**DOI:** 10.12688/f1000research.16405.2

**Published:** 2018-12-20

**Authors:** Indranath Chatterjee

**Affiliations:** 1 Department of Computer Science, University of Delhi, Delhi, 110007, India

**Keywords:** fMRI, Schizophrenia, Time-series, Classification

## Abstract

**Background: **Schizophrenia is a serious mental illness affecting different regions of the brain, which causes symptoms such as hallucinations and delusions. Functional magnetic resonance imaging (fMRI) is the most popular technique to study the functional activation patterns of the brain. The fMRI data is four-dimensional, composed of 3D brain images over time. Each voxel of the 3D brain volume is associated with a time series of signal intensity values. This study aimed to identify the distinct voxels from time-series fMRI data that show high functional activation during a task.

**Methods: **In this study, a novel mean-deviation based approach was applied to time-series fMRI data of 34 schizophrenia patients and 34 healthy subjects. The statistical measures such as mean and median were used to find the functional changes in each voxel over time. The voxels that show significant changes for each subject were selected and thus used as the feature set during the classification of schizophrenia patients and healthy controls.

**Results: **The proposed approach identifies a set of relevant voxels that are used to distinguish between healthy and schizophrenia subjects with high classification accuracy. The study shows functional changes in brain regions such as superior frontal gyrus, cuneus, medial frontal gyrus, middle occipital gyrus, and superior temporal gyrus.

**Conclusions: **This work describes a simple yet novel feature selection algorithm for time-series fMRI data to identify the activated brain voxels that are generally affected in schizophrenia. The brain regions identified in this study may further help clinicians to understand the illness for better medical intervention. It may be possible to explore the approach to fMRI data of other psychological disorders.

## Introduction

Schizophrenia is a severe mental disorder that affects different regions of the brain, often involving hallucinations and delusions. Functional magnetic resonance imaging (fMRI) data comprising 3D brain scans acquired over time (thus resulting in a 4D set) is often used to study brain regions affected by schizophrenia. Each voxel of the 3D brain volume is associated with a time series of signal intensity values. General linear model (GLM) ^1^ and independent component analysis (ICA) ^2^ are often employed to study the voxel activity by transforming the 4D time-series data to a 3D spatial map. 

The present work involves a novel application of mean deviation on time-series fMRI data to identify the distinct voxels that show high functional activation during a task. The work aims to identify the relevant brain regions that are affected in schizophrenia. Further, the identified voxels (features) are used to distinguish between schizophrenia patients and healthy subjects.

## Methods

### fMRI data

The time-series fMRI data having 1.5T strength was taken from the FBIRN phase – II data repository ^3^ available at site 0009 and site 0010. From the dataset, four different runs of auditory oddball task data of 34 schizophrenic patients (group G1) and 34 healthy controls (group G2) were extracted. Every run of each subject’s data contains 140 brain volumes acquired in 280 seconds time (TR = 2 seconds). Table 1 shows the dataset details.

**Table 1.** Dataset details.

Subject	Sample size	Age (Mean & Std Dev)	Sex (Male/Female)	Handedness (R/L)	Age of Onset (Median)	Smoking (Yes/No)
**Healthy**	34	37.76 (±12.25) years	24/10	30/4	NA	10/24
**Schizophrenia**	34	39.76 (±10.8) years	27/7	28/7	22 years	25/9

Pre-processing of the fMRI data was done using SPM8 toolbox in Matlab2014b. The temporal variation was corrected using slice timing correction, followed by the motion correction using realignment. Each of the fMRI scans was spatially normalized into standard Montreal Neurological Institute (MNI) space using an EPI template yielding voxel dimension of 3×3×3 mm ^3^. Finally smoothing was done using a 9×9×9 mm ^3^ full width at half maximum (FWHM) Gaussian kernel, resulting in a 3D brain volume containing 53×63×46, i.e., 1,53,594 voxels.

### Data analysis

The activation pattern of the voxels was analysed in two phases.

***Phase I.*** In the first phase, identification of voxels exhibiting high activation pattern (anytime during its time-course) is carried out for each subject. As the study focused on the variation in the signal intensity of the voxels ( **V**) over time, absolute mean deviation ( ${\overset{-}{\text{V}}}_{\text{d}}$) for each of the 140 time points was computed for each voxel, and the median ( *M*) of the 140 values of $\overset{-}{\text{V}}$ was found. Mean deviation ( $\overset{-}{\text{V}}$) values were compared with ***α*** times *M* ( ***α*** was chosen to be 3, based on experimentation) to identify whether a voxel exhibited high level of activation at any time during the 140 units of time. This voxel-wise analysis was performed for all the voxels of a given subject. Thus, a set of relevant voxels showing high degree of activation was obtained for each subject.

***Phase II.*** In the second phase, a common subset of voxels exhibiting high degree of activation across all the subjects within a group was obtained. Finally, both the subsets belonging to groups G1 (schizophrenia patients) and G2 (healthy controls) were merged to get the set *S*. The voxels in set *S* were backtracked to MNI brain space and finally mapped into Talairach’s space ^4^ to identify the brain regions. This procedure has been described in Algorithm 1.

***Classification.*** The set *S* was used to distinguish between schizophrenia patients and healthy subjects using two classifiers, viz., support vector machine (SVM) with sigmoid kernel ^5^ and extreme learning machine (ELM) classifier ^6^.

***Experimental settings.*** All the implementations were done in MATLAB2014b. Parameter ***α*** was varied in the range of 1 to 7 in steps of 1 to identify the number of voxels that exhibited a high level of activations during the task. When the value of α was taken as 1 and 2, a large number of voxels showed activation level higher than ***α*** times *M*, resulting in set *S* having voxels that represents almost the entire brain. However, for ***α*** **=** 3, it was found that set *S* contained only 1580 distinct voxels that mapped to the brain regions which are generally affected in schizophrenia. When ***α*** was taken as more than 3, the number of voxels in the set *S* were close to zero rendering it too small for any meaningful analysis. Thus, ***α*** = 3 was found to be the most suitable value.

Further, the set *S* of voxels obtained ***α*** = 3 was used to fine-tune the classifiers. The SVM classifier gave the best results for the regularization parameter *C* = 1.09, and sigmoid kernel based ELM classifier gave best the results with 503 hidden neurons. 

To evaluate the distinguishing capability of the voxels/features in set *S*, a comparison was done between the classification accuracy obtained using *S* and the accuracy obtained using the voxels set given by the GLM based approach. In this case, GLM was applied using SPM8 toolbox to convert the 4D time-series fMRI data to 3D contrast map for each subject. The GLM yielded an activation map comprising around 60000 voxels out of 153594 which were activated during the task.

**Algorithm 1. The proposed approach**

*Notations*:

*m* (=34): the number of subjects in each group
*n* (=140): the number of observations in a run
**V *_i_*** : time-series of *i* ^th^ voxel
i.e. **V *_i_*** = [ *v _i,1_* *v _i,2_* *v _i,3_* ⋯ *v _i,n_*];
$\mu_{i}\;:\;\mathrm{mean}\;\mathrm{of}\;V_{i}\;\mathrm{i.e.}\;\mu_{i}\;=\;\frac{{\sum_{j\;=\;1}^{j\;=\;n}v}_{i,j}}{n}$

*Steps*:

1. Calculate absolute mean deviation for each voxel using ${\overset{-}{V}}_{d_{i}}=\left|{\text{V}}_{i}-\mu_{\iota }\right|.$
2. Find median *M _i_* of ${\overset{-}{V}}_{d_{i}}.$
3. For each subject *k* ∈{1,2,..., *m*}, select the set ***V _s _k__*** of voxels that show deviation higher than *αM _i_*.
4. Find the group wise intersection of the voxels selected in step 3 for groups *G*1 and *G*2
  i.e. $V_{s_{G1}}\;=\;\cap_{k=1}^{m}\;V_{s_{k}}\;(G1)$
$\;V_{s_{G2}}\;=\;\cap_{k=1}^{m}\;V_{s_{k}}\;(G2)$
5. Merge the two sets, obtained in step 4 to obtain set ***S***
  i.e. ***S*** = ***V*_*S*_*G*1__** ∪ ***V*_*S*_*G*2__**
6. Map ***S*** into the brain space to identify affected regions.

## Results

**Figure 1.**
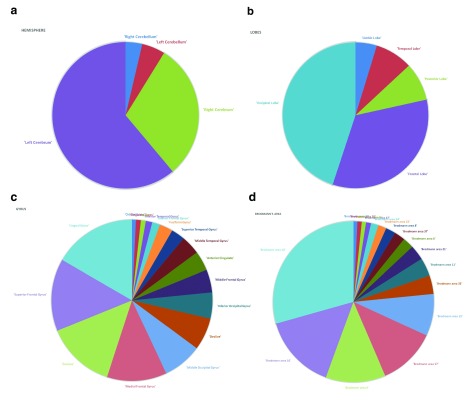
Identified brain regions at different levels of hierarchy, namely, hemisphere level ( **a**), lobes level ( **b**), gyrus level ( **c**), and Brodmann’s area level ( **d**).

**Figure 2.**
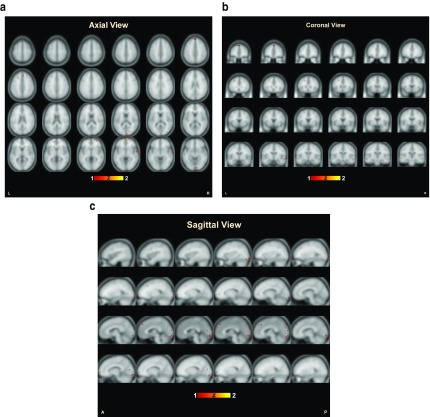
Voxels identified by the proposed approach plotted over a functional brain image in different views of the brain, i.e., axial ( **a**), coronal ( **b**) and sagittal ( **c**) plane.

A comparison of the results of the classification accuracies obtained using feature sets given by the GLM and the proposed approach is shown in Table 2. The features selected by the proposed approach when backtracked to Talairach’s space revealed the brain regions that are generally affected in schizophrenia ^7– 9^, which validates the efficacy of the approach. The distribution of the selected voxels that distinguish the schizophrenia patients from the healthy subjects is shown in Figure 1 (a–d). The results show the increased changes in functional activation in the regions such as occipital lobe, frontal lobe, posterior lobe, and temporal lobe. When looking into the level of gyri, certain changes in activation pattern are seen in superior temporal gyrus, lingual gyrus, cuneus, declive, medial frontal gyrus, and middle occipital gyrus. Some regions in Brodmann areas (BA 18, 10, 9, 17, 19, 32, 6, 37, 21, 22, 46, and 47) also show distinct changes in functional activation in schizophrenics when compared to healthy controls. Figure 2 (a–c) show the activated voxels when plotted on a sample fMRI image for an axial, coronal and sagittal view of the brain.

**Table 2.** Comparison showing classification accuracy with feature set obtained after GLM and the proposed approach using SVM and ELM classifiers.

	GLM	Proposed approach
**Number of voxels**	~ 60,000	~ 1580
**SVM with Sigmoid kernel**	32.45%	76.47%
**ELM with Sigmoid kernel**	57.35%	61.46%

GLM, general linear model; SVM, support vector machine; ELM, extreme learning machine.

## Discussion

Unlike other conventional methods such as GLM to select the voxels showing a statistically significant response to the experimental conditions ^10^, the proposed approach identifies the neural activity in a particular voxel with the help of bold signal over time, irrespective of any experimental condition. The proposed approach does not require any details for the task and conditions. It works on the temporal values of each voxel for each subject's data one by one. Like other multi-voxel pattern analysis (MVPA) methods ^10– 12^, this approach also tries to find the participation of multiple voxels when selecting the final set of relevant voxels across a particular group of the subjects.

The classification accuracies, as shown in Table 2, demonstrate the efficacy of the proposed methodology. The reduced set of 1580 voxels achieved a much higher accuracy when compared to the GLM approach. The approach gives a better result for both of the SVM and ELM classifiers when compared to the GLM approach.

Figures 1 (a–d) show the distribution of the selected voxels for each level of brain regions. These regions show distinct changes in functional activation in schizophrenia patients when compared to healthy controls, and thereby distinguish between schizophrenia and healthy subjects with high classification accuracy. Most of the regions identified in the study comply with the existing literature ^13– 16^.

From the Figure 1 (b), change in functional activation can be seen in the frontal lobe which is responsible for motor function, executive functions and attention ^17, 18^. Literature ^19, 20^ suggest frontal lobe functional dysfunction in schizophrenia. Significant changes are observed in the temporal lobe and occipital lobe as seen in Figure 1 (b). The temporal lobe is basically responsible for holding primary auditory perception such as hearing, and occipital lobe is responsible for visual perception. As schizophrenia patients suffer from auditory and visual hallucinations, the functional deficit in these regions are responsible for the symptoms, studies ^21, 22^ also suggest functional changes in these areas in schizophrenia.

As seen in Figure 1 (c), significant changes in functional activations are found in regions such as superior frontal gyrus, superior temporal gyrus, lingual gyrus, medial frontal gyrus, middle occipital gyrus, anterior cingulate, cuneus, and declive. Superior temporal gyrus contains the primary auditory cortex which is responsible for processing sound, sending sensory information to auditory cortex and also to specify the sound frequencies precisely. Previous studies ^23– 25^ also showed that the superior temporal gyrus gets affected in schizophrenia. Figure 1 (c) shows functional changes in superior frontal gyrus, which is mainly involved in self-awareness ^26^. Literature ^13, 27^ suggests changes in superior frontal gyrus. The literature also suggests functional changes in middle occipital gyrus ^28^. Since schizophrenia patients also suffer from visual hallucinations and deficiency in visual attention, the dysfunctioning of the areas such as declive and cuneus (BA 17) may play role in the disorder. Studies found that cuneus ^15, 29^ and declive ^30^ show functional changes in schizophrenia. Lingual gyrus is basically linked to function for visual processing ^31^. The result of this study also shows subtle functional changes in lingual gyrus indicating difficulties in visual abilities in schizophrenia ^32, 33^. Even functional abnormality in anterior cingulate was found in several studies ^16, 34^.

In the level of Brodmann’s area, as seen in Figure 1 (d), BA 18 and 19 show functional changes in schizophrenia patients when compared to healthy controls. These regions lie in the occipital cortex, mainly responsible for the interpretation of images ^35^. Studies ^36, 37^ show that these regions are commonly affected in schizophrenia. BA 10, lies in the prefrontal cortex, is responsible for executive functions such as attention, processing of working memory and taking decision for future actions ^38^. Similar to the previous studies ^39, 40^, this study also identifies the functional changes in BA 10. BA 9 lies in the frontal lobe, mainly responsible for short-term memory, auditory verbal attention. This region may play a vital role in auditory hallucination and in retrieving the short-term memory in schizophrenia patients ^41, 42^. BA 37, responsible for visual fixation ^43^ and recognizing true-false memory, is also mentioned in the previous studies ^44, 45^. BA 21 and 22 lie in the temporal cortex, believed to play a role in auditory processing are found to be affected in schizophrenia ^19, 20^. Other than these regions, the result shows significant changes in functional activations in the areas such as BA 6, BA 37, BA 46, and BA 11, which are also reported in the literature ^14, 46, 47^.

This paper identifies the affected brain regions in schizophrenia and compares them with the previous studies. As the study focused on the statistical measures derived from the voxel values across the time course, the effect of covariates such as level of education, duration of disease, and medication history could not be incorporated. However, during the group-wise analysis (mentioned in Phase II of the methodology), a grouping of subjects’ data was also performed on the basis of gender and age. The results obtained were quite uniform across all ages and genders. Although this study was performed on the auditory oddball task fMRI data, it would be interesting to explore the applicability of the approach on the resting-state fMRI data and other performance-based tasks.

## Conclusions

This work describes a simple and fast feature selection algorithm based on mean deviation for time-series fMRI data to identify the activated brain voxels that are generally affected in schizophrenia. The proposed approach was found to be efficient in selecting a minimal set of relevant voxels directly from time-series 4D fMRI data. The obtained voxel set was capable of distinguishing between healthy and schizophrenic subjects. One may explore the possibility of applying this approach to fMRI data of other psychological disorders.

## Data availability

The data referenced by this article are under copyright with the following copyright statement: Copyright: © 2018 Chatterjee I

Data associated with the article are available under the terms of the Creative Commons Zero "No rights reserved" data waiver (CC0 1.0 Public domain dedication). 

The Matlab source codes, a text file containing dataset details including subject ID and their age, and the instructions for the study can be found at: https://github.com/IndraChatterjee/AnomalyDetection_TimeSeries_fMRI_Schizophrenia.

The complete source codes are archived in a publicly accessible record at: https://doi.org/10.5281/zenodo.1438539 ^48^

License: CC0

The four runs of auditory oddball task fMRI data from the FBIRN phase II repository can be downloaded from http://schizconnect.org/ querying 1.5T fMRI data for healthy and schizophrenia subjects available at site 0009 and 0010. The list of subjects chosen for this study is mentioned in the ‘DataDetails_FBIRN15T.txt’ file available at the GitHub repository. Users are required to sign-up to SchizConnect to download data and conditions of use are as written in the data use agreement of the FBIRN project.

## Author endorsement

Cameron Craddock confirms that the author has an appropriate level of expertise to conduct this research, and confirms that the submission is of an acceptable scientific standard. Cameron Craddock declares the following competing interests: I am the Chair of Brainhack, and this organisation awarded this paper this year's Brainhack poster prize. Affiliation: Associate Professor of Diagnostic Medicine, Dell Medical School, The University of Texas at Austin, Austin, TX, USA.
