# Supplementary material for: Mean deviation based identification of activated voxels from time-series fMRI data of schizophrenia patients
Source: F1000Res. 2018 Dec 20;7:1615. Originally published 2018 Oct 8. [Version 2] doi: 10.12688/f1000research.16405.2 (PMC6338245; doi:10.12688/f1000research.16405.2)
Supplement: Peer review history

# Peer review: Referee response for version 2

**DOI:** 10.5256/f1000research.19127.r42164

**Published:** 2019-01-16

**Authors:** Sagarika Bhattacharjee

**Affiliations:** 1 Department of Psychology, Nanyang Technological University, Singapore, Singapore

I thank the author for making the revision.

I have read this submission. I believe that I have an appropriate level of expertise to confirm that it is of an acceptable scientific standard.

# Peer review: Referee response for version 1

**DOI:** 10.5256/f1000research.17921.r39217

**Published:** 2018-11-22

**Authors:** Sagarika Bhattacharjee

**Affiliations:** 1 Department of Psychology, Nanyang Technological University, Singapore, Singapore

The present study describes an methodology that classifies schizophrenia patients from healthy controls.

 The study claims to demonstrate high classification accuracy.

 The study has significant relevance to the neuroscience community however I have following concerns:

1. The functional significance of the brain regions involved needs to be elaborated so that their activation could be validated. The description of the role of obtained brain regions in schizophrenia patients and healthy individuals will indicate that the obtained regions are actually involved in schizophrenic patients and not a result of Type II error.
2. It will be good to provide some details about the demographics, level of education, duration of disease, medication history of the participants in order to evaluate the role of these confounding factors in the obtained results. These factors might cause some variation in the fMRI signals and just wondering if any of these parameters were taken as covariate in the analysis.
3. The fMRI signals obtained are task state and not resting state. These signals were obtained while doing oddball paradigm. So, I was wondering whether such classification would apply to schizophrenia patients only when they are doing this particular task, or it would apply to all schizophrenic patients irrespective of their state.

I have read this submission. I believe that I have an appropriate level of expertise to confirm that it is of an acceptable scientific standard, however I have significant reservations, as outlined above.

# Response

**Published:** 2018-12-09

**Authors:** Indranath Chatterjee

**Affiliations:** University of Delhi, India

I am thankful to the respected referee for her insightful comments and valuable suggestions.

 I have updated the manuscript in accordance with the suggestions and queries. The suggested changes are made in the discussion section of the revised manuscript. I have also included some demographic details of the subjects in the dataset table. As I have not incorporated any covariates in this study, the limitation and the scope of future works are added in the discussion section.

# Peer review: Referee response for version 1

**DOI:** 10.5256/f1000research.17921.r39310

**Published:** 2018-11-01

**Authors:** Sahil Bajaj

**Affiliations:** 1 Social, Cognitive and Affective Neuroscience Laboratory (SCAN Lab), Department of Psychiatry, College of Medicine, University of Arizona, Tucson, AZ, USA

Here author describes an interesting algorithm to identify the activated brain voxels affected in schizophrenia from time-series fMRI data.

 I think this is an interesting paper and a nice example which can be implemented in more severe cases of mental distress.

 However, I have few minor concerns:

-  How did the author remove the effect of gender, I noticed that there are way more males in the data than females.
- I can see some voxels outside the brain and which are at the skull. I am not sure why did the author get those voxels and if the author made any effort to exclude those voxels?

I have read this submission. I believe that I have an appropriate level of expertise to confirm that it is of an acceptable scientific standard.
